# Impact of chronic hypoxia on proximal pulmonary artery wave propagation and mechanical properties in rats

**DOI:** 10.1152/ajpheart.00695.2017

**Published:** 2018-03-16

**Authors:** Junjing Su, Charmilie C. Logan, Alun D. Hughes, Kim H. Parker, Niti M. Dhutia, Carl Christian Danielsen, Ulf Simonsen

**Affiliations:** ^1^Department of Biomedicine, Aarhus University, Aarhus, Denmark; ^2^Institute of Cardiovascular Science, University College London, London, United Kingdom; ^3^Department of Bioengineering, Imperial College London, London, United Kingdom

**Keywords:** arterial stiffness, extracellular matrix, hypoxia, pulmonary hypertension, pulse wave velocity

## Abstract

Arterial stiffness and wave reflection are important components of the ventricular afterload. Therefore, we aimed to assess the arterial wave characteristics and mechanical properties of the proximal pulmonary arteries (PAs) in the hypoxic pulmonary hypertensive rat model. After 21 days in normoxic or hypoxic chambers (24 animals/group), animals underwent transthoracic echocardiography and PA catheterization with a dual-tipped pressure and Doppler flow sensor wire. Wave intensity analysis was performed. Artery rings obtained from the pulmonary trunk, right and left PAs, and aorta were subjected to a tensile test to rupture. Collagen and elastin content were determined. In hypoxic rats, proximal PA wall thickness, collagen content, tensile strength per unit collagen, maximal elastic modulus, and wall viscosity increased, whereas the elastin-to-collagen ratio and arterial distensibility decreased. Arterial pulse wave velocity was also increased, and the increase was more prominent in vivo than ex vivo. Wave intensity was similar in hypoxic and normoxic animals with negligible wave reflection. In contrast, the aortic maximal elastic modulus remained unchanged, whereas wall viscosity decreased. In conclusion, there was no evidence of altered arterial wave propagation in proximal PAs of hypoxic rats while the extracellular matrix protein composition was altered and collagen tensile strength increased. This was accompanied by altered mechanical properties in vivo and ex vivo.

**NEW & NOTEWORTHY** In rats exposed to chronic hypoxia, we have shown that pulse wave velocity in the proximal pulmonary arteries increased and pressure dependence of the pulse wave velocity was steeper in vivo than ex vivo leading to a more prominent increase in vivo.

## INTRODUCTION

Pulmonary arterial hypertension (PAH) is a progressive disease characterized by vasoconstriction, vascular remodeling, inflammation, and thrombosis (8), resulting in increased pulmonary vascular resistance (PVR), vessel stiffening, and vascular impedance mismatch, leading to the generation of large reflected waves (30, 46, 57). All of the above contribute to increased right ventricular (RV) afterload and, ultimately, RV failure. Despite advances in pharmacological treatment, mortality from PAH remains high, with a 3-yr survival rate of only 58% (52). PAH-specific drugs, such as phosphodiesterase-5 inhibitors, prostanoid analogs, and endothelin receptor antagonists, mainly target the pulmonary arterioles and aim to decrease PVR, i.e., the steady flow component of RV afterload (52). However, it has become apparent in recent years that arterial stiffness is independently associated with RV dysfunction (55) and is a strong independent predictor of mortality in PAH (34).

Commonly, the proximal pulmonary arteries (PAs), i.e., the pulmonary trunk (PT) and right and left PAs, are considered to be capacitance vessels that determine the pulsatile component of RV afterload (26). Several ex vivo studies have documented substantial proximal PA stiffening with increased collagen content in widely used experimental pulmonary hypertension models: the hypoxic rodent and bovine models (25, 27, 68). However, whether the mechanical properties ex vivo match the in vivo conditions and whether hypoxia also affects the mechanical properties of the aorta have not been well elucidated.

Arterial pulse wave transmission is also an important component of ventricular load. Four types of arterial waves have been described in the pulmonary artery. Forward compression waves (which increase pressure and flow) and forward decompression waves (which decrease pressure and flow) are related to ventricular contraction and relaxation, respectively. Backward compression waves (which increase pressure while decreasing flow) and backward decompression waves (which decrease pressure while increasing flow) are reflected waves due to vascular impedance mismatch between the proximal and distal vasculature (56). Pulse wave velocity (PWV), i.e., the speed at which a wave travels through an artery, is an indicator of arterial stiffness. Analyses of arterial waves include traditional vascular impedance analysis and the more recently developed wave intensity (WI) analysis (WIA). Such analyses generally require simultaneous measurement of blood pressure and flow, and, due to small body size of the animals, they are rarely performed in rodents (50, 51, 60).

In this study, we performed a comprehensive ex vivo assessment of the mechanical properties of proximal PAs and the aorta in the hypoxic rat model. In addition, we explored the feasibility of invasive pulmonary WIA and investigated how in vivo measurements of arterial stiffness relate to ex vivo measurements.

A glossary for the variables and abbreviations used in this report is shown in Table 1.

**Table 1.** Glossary

	Definition
*A*	Vessel wall area
CCD	Cardiac cycle duration
*d*	Vessel luminal diameter
*d*_d_	Diastolic pulmonary trunk diameter
*d*_h_	Diameter of the hooks
*d*_s_	Systolic pulmonary trunk diameter
*D*	Distensibility
*E*	Incremental elastic modulus
*E*_p_	Elastic modulus at physiological distending pressures
ECM	Extracellular matrix
F	Force
GAG	Glycosaminoglycan
*h*	Vessel wall thickness
*k*	Incremental stiffness
*l*	Vessel length
*l_c_*	Luminal circumference
*l_c_*,_0_	Initial luminal circumference
*l_l_*	Longitudinal length
P	Pressure
P_b_	Backward pressure waveform
P_f_	Forward pressure waveform
PA	Pulmonary artery
PAH	Pulmonary arterial hypertension
PAP_m_	Mean pulmonary arterial pressure
PAP_p_	Pulmonary arterial pulse pressure
PT	Pulmonary trunk
PVR	Pulmonary vascular resistance
PWV	Pulse wave velocity
PWV_BH_	Pulse wave velocity calculated using the Bramwell and Hill equation
PWV_MK_	Pulse wave velocity calculated using the modified Moens-Korteweg equation
PWV_sq_	Pulse wave velocity calculated using the sum of squares method
*r*	Vessel luminal radius
RHC	Right heart catheterization
RV	Right ventricle/right ventricular
*t*	Time
T	Vessel wall tension
TTE	Transthoracic echocardiography
*U*	Velocity
VTI	Velocity time integral
WI	Wave intensity
WI_+_	Forward component of wave intensity
WI_−_	Backward component of wave intensity
WIA	Wave intensity analysis
WRI	Wave reflection index
*x*	Hook distance
ε	Strain
ν	Poisson’s ratio
ρ	Blood density
σ	Stress

## MATERIALS AND METHODS

### 

#### Experimental design.

Experiments were performed in accordance with European Union Directive 2010/63 and approved by the Animal Experiments Inspectorate (permission no. 2012-15-2934-00741). A total of 48 male Sprague-Dawley rats (10 wk, ∼350 g, Taconic Biosciences, Lille Skensved, Denmark) were randomly assigned to 2 groups (24 animals/group). One group was exposed to hypoxia by being placed in hypobaric chambers (560 mbar corresponding to 10% oxygen) (6). The chambers were ventilated with air (45 l/min), and the temperature was maintained at 20°C. Two times per week, the chambers were opened for a maximum of 30 min for maintenance purposes. Rats in the control group were placed in similar but normoxic and normobaric chambers.

After 21 days, 12 animals from each group were subjected to in vivo hemodynamic measurements as described below. The remaining animals were euthanized by decapitation. The descending thoracic aorta, PT, and right and left PAs were harvested for histological examination or stored at −20°C until used for mechanical testing.

#### Hemodynamic measurements.

At the end of the 21-day period, transthoracic echocardiography (TTE; Vevo2100, MS250 linear array probe, Visual Sonics, Toronto, ON, Canada) was performed on a spontaneously breathing rat anesthetized with sevoflurane (induction: 7% and maintenance: 4%) in oxygen. B-mode and pulsed wave Doppler images of the PT were acquired (1).

Subsequently, anesthetized animals (sevoflurane in a 3:1 mixture of oxygen and nitrous oxide) were ventilated at 75 strokes/min with a tidal volume of 10 ml/kg (model 7025, Ugo Basile, Varese, Italy). Right heart catheterization (RHC) was performed through a left lateral thoracotomy. A 0.014-in. combined dual-tipped pressure and Doppler flow sensor wire (Combowire, Philips Volcano) was inserted through the RV outflow tract into the PT to acquire pressure and velocity data at a sampling rate of 200 Hz (Combomap, Philips Volcano). At the end of the procedure, animals were euthanized by decapitation.

#### Hemodynamic calculations.

RV stroke volume (*Eq. 1*) was calculated using the velocity time integral (VTI) and the systolic PT diameter (*d*_s_) obtained by TTE as follows:

(1) $$\text{RV}\;\text{stroke}\;\text{volume}=\pi \times {\left(0.5\times d_{\text{s}}\right)}^{2}\times \text{VTI}$$

By neglecting right atrial pressure, RV stroke work was defined as the product of RV stroke volume and mean pulmonary arterial pressure (PAP_m_). Total pulmonary resistance was calculated as PAPm divided by cardiac output, and global pulmonary arterial compliance was calculated as stroke volume divided by pulmonary arterial pulse pressure (PAP_p_). Using diastolic PT diameter (*d*_d_) and arterial distensibility (*D*; *Eq. 2*) and assuming a blood density (ρ) of 1,040 kg/m^3^, the local pulse wave velocity (PWV_BH_, *Eq. 3*) was calculated using the Bramwell and Hill equation (5), as follows:

(2) $$D=\frac{d_{\text{s}}^{2}-d_{\text{d}}^{2}}{d_{\text{d}}^{2}\times {\text{PAP}}_{\text{p}}}$$

(3) $${\text{PWV}}_{\text{BH}}=\frac{1}{\sqrt{\rho \times D}}$$

Using ensemble-averaged pulmonary arterial pressure and velocity obtained by RHC, PWV_sq_ was calculated using the sum of squares method (*Eq. 4*) (13), where the sum was taken over one cardiac period, as follows:

(4) $${\text{PWV}}_{\text{sq}}=\frac{1}{\rho }\times \sqrt{\frac{\Sigma {\text{dP}}^{2}}{\Sigma \text{d}U^{2}}}$$

where P is pressure and U is velocity.

Pulmonary WIA was performed using the ensemble-averaged pressure and velocity obtained by RHC as well as RHC-derived pressure combined with TTE-derived velocity. Given the cardiac cycle duration (CCD), WI (*Eq. 5*) was normalized to the number of samples squared in the cardiac cycle and separated into its forward (WI_+_) and backward (WI_−_) components (*Eq. 6*) (58). The measured pressure was separated into forward (P_f_) and backward (P_b_) pressure waveforms by integrating the differentials, dP_f_ and dP_b_ (*Eq. 7*) (22), as follows:

(5) $$\text{WI}=\frac{\text{dP}\times \text{CCD}}{\text{d}t}\times \frac{\text{d}U\times \text{CCD}}{\text{d}t}$$

(6) $${\text{WI}}_{\pm }=\pm {\left(\frac{\text{dP}}{\text{d}t}\pm \rho \times {\text{PWV}}_{\text{sq}}\times \frac{\text{d}U}{\text{d}t}\right)}^{2}\times {\text{CCD}}^{2}/\left(4\rho \times {\text{PWV}}_{\text{sq}}\right)$$

(7) $$\frac{{\text{dP}}_{\text{f/b}}}{\text{d}t}=\frac{1}{2}\times \left(\frac{\text{dP}}{\text{d}t}\pm \rho \times \frac{\text{d}U}{\text{d}t}\times {\text{PWV}}_{\text{sq}}\right)$$

The separated waves were quantified by their peak intensity (power density) and energy density over a cardiac cycle squared. Wave reflection was quantified as the wave reflection index (WRI), defined as the ratio of backward to forward wave energy and as the P_b_-to-P_f_ ratio.

Details on pressure and velocity data processing can be found in the appendix.

#### Mechanical testing.

**Fig. 1.**
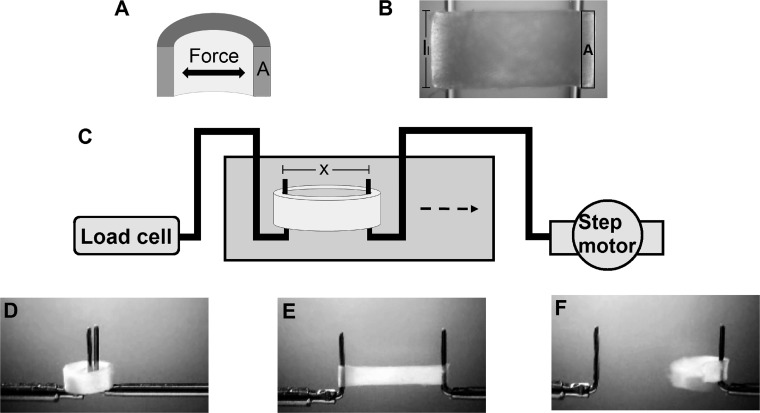
Overview of the mechanical testing setup. Uniaxial mechanical testing was performed on isolated artery rings, where two sides of the artery ring (*A*; schematic sagittal section) corresponding to two vessel wall area (outlined in black) were subjected to a given load simultaneously. *B*: before mechanical testing, the artery ring segments with a longitudinal length (*l_l_*) of 1 mm were mounted on a glass rod and photographed to measure the vessel wall area (outlined in black). *C*: the artery ring was then mounted around two bent steel hooks, which were connected to a load cell and step motor, respectively (schematic overview), and subjected to a tensile test to rupture (*D–F*). See Table 1 for abbreviations.

Frozen aortas and PAs were cut into one to three rings with a nominal longitudinal length of 1 mm. Excess artery tissues were set aside for the determination of extracellular matrix (ECM) protein composition. Subsequently, artery rings were mounted on a tapered glass rod at minimal strain and photographed (Fig. 1) using a microscope (Nikon, Tokyo, Japan) with a circular polarization filter (62). The vessel wall area that would be subjected to loading was then traced using ImageJ (v1.51, National Institutes of Health) and calibrated using a photograph of a millimeter scale. Afterward, artery rings were stored in calcium- and magnesium-free 50 mM Tris·HCl buffer solution (pH 7.4) at −20° until further use.

Once thawed at room temperature, artery rings were subjected to mechanical testing (Fig. 1) using a custom-designed setup (62). Each ring was mounted around two parallel, orthogonally bent steel hooks submerged in Tris·HCl solution. One hook was connected to a load cell (Kistler-Morse DSC-6 transducer, DMT, Aarhus, Denmark), and the other hook was connected to a step motor (DM224i, API Motion, New York, NY) that moved at a constant speed of 0.167 mm/s. Load cell readings and the moveable hook’s travel distance were continuously acquired by a data-acquisition unit (model 34970A, Hewlett Packard) and from the step motor drive, respectively. For preconditioning, each artery ring was subjected to five loading-unloading cycles with a maximum load of 25 mN for the aorta, 15 mN for the PT, and 10 mN for right and left PAs. Afterward, a stretch test to rupture was carried out, and ruptured artery rings were collected for hydroxyproline determination.

#### Calculation of mechanical properties.

The luminal circumference (*l_c_*; *Eq. 8*) of each artery ring was calculated based on the distance between the distant hook surfaces (*x* = distance at starting position + travel distance) and the diameter of the hooks (*d*_h_ = 0.35 mm). The initial luminal circumference (*l_c_*,_0_) was calculated at a minimal loading force (0.1 mN) as follows:

(8) $$l_{c}=2\times \left(x-d_{\text{h}}\right)+\left(\pi \times d_{\text{h}}\right)$$

Two sides of the artery ring corresponding to the double vessel wall area were loaded simultaneously (Fig. 1). Thus, all calculations were based on half of the load [force (F)] applied to one vessel wall area (*A*). Assuming that the vessel wall was incompressible and homogeneous and neglecting the residual stress, strain (ε), stress (σ), and the incremental stiffness (*k*) and elastic modulus (*E*) were calculated using *Eqs. 9**−**12* as follows:

(9) $$\varepsilon =\frac{\Delta l_{c}}{l_{c,0}}$$

(10) $$\sigma =\frac{\text{F}}{A}$$

(11) $$k=\frac{\text{dF}}{\text{d}\varepsilon }$$

(12) $$E=\frac{\text{d}\sigma }{\text{d}\varepsilon }=\frac{k}{A}$$

The maximum stiffness and maximum elastic modulus were defined as maximum dF/dε and dσ/dε, respectively.

To investigate the mechanical properties of each artery ring at physiologically relevant loads, distending pressures equivalent to pressures of 80, 100, and 120 mmHg were used for the aorta and 11, 16, and 24 mmHg for proximal PAs. Given a longitudinal length (*l_l_*), luminal radius (*r*), and vessel wall thickness (*h*), the distending pressures (P), wall tension (T), wall stress (σ_wall_), and corresponding strains were derived from the load-strain curves by estimating the combination of load and strain that fulfilled Laplace’s law for a cylinder (*Eqs. 13* and *14*) (62). The stiffness and elastic modulus (*E*_p_) at each of the three distending pressures were calculated as described above (*Eqs. 11* and *12*):

(13) $$\text{T}=\frac{\text{F}}{l_{l}}=\text{P}\times r↔\text{P}=\frac{\text{F}}{l_{l}\times r}$$

(14) $$\sigma_{\text{wall}}=\frac{\text{T}}{h}$$

Assuming that the vessel wall is nearly incompressible with a Poisson’s ratio (ν) of 0.45 (39), the modified Moens-Korteweg equation was used to calculate PWV (PWV_MK_; *Eq. 15*) at each distending pressure as follows:

(15) $${\text{PWV}}_{\text{MK}}=\sqrt{\frac{E\times h}{2r\rho \times \left(1-\nu^{2}\right)}}$$

Finally, from the last hysteresis cycle, the viscous energy loss was calculated as the area inside the hysteresis loop divided by the area under the load-deformation curve.

#### Determination of ECM components.

The hydroxyproline content of each artery ring was quantified by colorimetric determination through a modified Woessner method (12, 69). Collagen content was calculated based on the assumption that hydroxyproline forms 13.4% of total collagen (36). Assuming that collagen is responsible for the ultimate strength of the vessel wall, the mechanical quality of collagen was assessed by its maximal tensile strength defined as the maximum loading force per unit collagen.

Excess artery tissues were gathered into several sample pools and defatted with acetone before quantification of the fractional elastin and collagen content. Mature elastin was purified through the hot alkali method (28). After centrifugation, the supernatant was removed and the residue that remained was assumed to be elastin; its dry weight was expressed as a percentage of the defatted dry weight. Hydroxyproline content in the extract was determined as described above.

The glycosaminoglycan (GAG) content (sulfated + nonsulfated) in the extract was estimated by quantifying the uronic acid concentration through colorimetric determination as previously described (4). The amount of sulfated GAG was estimated by the 1,9-dimethylmethylene blue procedure (3).

#### Histology.

The aorta and PAs were fixed at zero transmural pressure in 4% formaldehyde overnight and subsequently preserved in 70% ethanol. Vessels were then embedded in paraffin and sectioned into 4-μm-thick slices. Selected sections from each rat were stained with either hematoxylin and eosin or with resorcin (Hart’s solution), Sirius red F3B, and Weigert’s hematoxylin.

#### Statistical analysis.

Data are presented as means ± SD (SE is used for graphical presentation) or medians (25–75% quartiles). Statistical comparison of hemodynamic parameters within each group was performed using a paired Student’s *t*-test. Differences between the normoxic and hypoxic groups were compared using an unpaired Student’s *t*-test or Welch’s *t*-test for unequal variances. Parameters derived from the mechanical testing were summarized taking clustered data into account, and comparisons within each group as well as between the two groups were performed using modified rank tests for clustered data (37). The level of significance was set at *P* < 0.05. All statistical analyses were performed using Stata (version 13, StataCorp).

## RESULTS

### 

#### Hemodynamics.

Three rats died during the invasive procedure. Hence, 22 normoxic rats and 23 hypoxic rats were included in the study. The effect of hypoxia was evidenced by the lower weight gain, shorter tibia length, higher hematocrit, and higher Fulton’s index (RV weight/left ventricle + septum weight) in the hypoxic group (Table 2).

**Table 2.** Physical characteristics of the animals

	Normoxia	Hypoxia	*P* Value
*n*	22	23	
Body weight at end point, g	412 ± 25	374 ± 24	<0.01*
Weight gain, g	51 ± 17	22 ± 13	<0.01*
Hematocrit, %	44.3 ± 6.5	56.6 ± 5.6	<0.01*
Lungs, g†	1.7 ± 0.2	2.4 ± 0.2	<0.01*
Right ventricle, g	0.23 ± 0.04	0.30 ± 0.07	<0.01*
Left ventricle + septum, g‡	0.91 ± 0.10	0.76 ± 0.08	<0.01*
Fulton index	0.25 ± 0.02	0.39 ± 0.07	<0.01*
Liver, g‡	14.1 ± 2.4	13.5 ± 1.9	0.36
Spleen, g‡	0.89 ± 0.12	0.83 ± 0.11	0.08
Tibia length, cm	4.1 ± 0.1	4.0 ± 0.1	0.01*

Values are presented as means ± SD; *n*, number of animals/group.

* *P* < 0.05;

† *n* = 12 animals/group;

‡ Left ventricular weight remained significantly lower and liver and spleen weight remained insignificantly different after indexing them to body weight and tibia length.

**Fig. 2.**
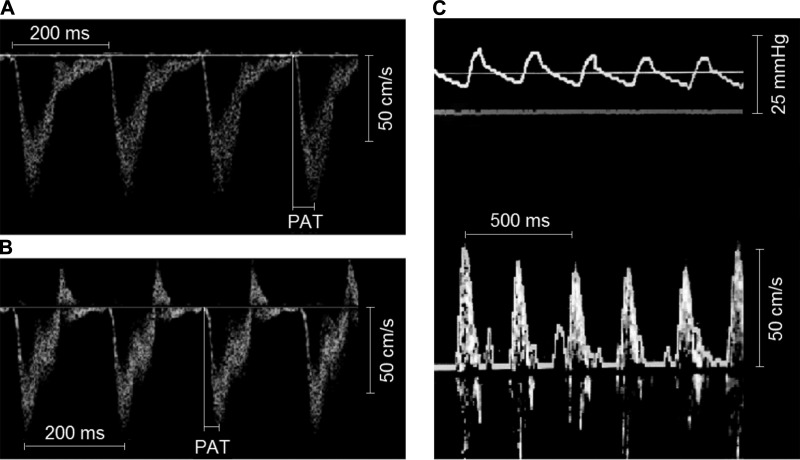
Representative flow velocity traces from the pulmonary trunk. Noninvasive (transthoracic echocardiography) pulsed-wave Doppler flow traces from the pulmonary trunk are shown for a normoxic rat (*A*) and a hypoxic rat (*B*). A midsystolic notch during the flow deceleration phase and shorter pulmonary acceleration time (PAT) were observed in hypoxic animals. Trivial diastolic flow reversal was observed in the majority of the traces (both normoxic and hypoxic animals). *C*: the corresponding invasive (pulmonary artery catheterization) pressure measurement (*top*) and Doppler flow trace (*bottom*) with velocity tracking from the same normoxic rat.

**Fig. 3.**
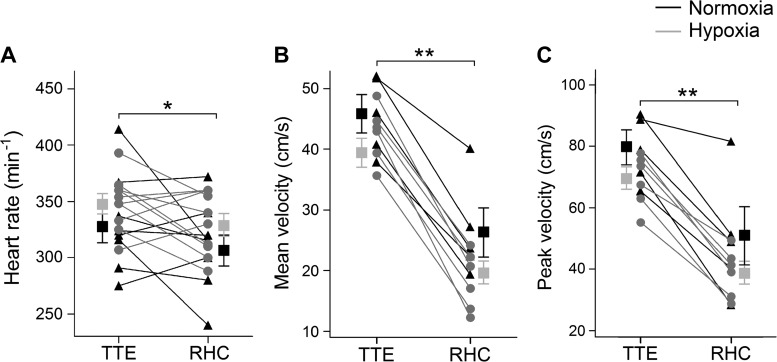
Invasive versus noninvasive measurements. *A−C*: lower heart rate (*A*) and mean and peak pulmonary flow velocities (*B* and *C*, respectively) were observed during right heart catheterization (RHC; performed in the open-chested condition) compared with measurements acquired by transthoracic echocardiography (TTE; performed in the close-chested condition). Squares and error bars represent means ± SE. *P < 0.05; **P < 0.01.

Representative pressure and flow velocity traces from the PT are shown in Fig. 2, and hemodynamic data are shown in Table 3. A midsystolic notch was observed in the Doppler flow envelope obtained by TTE but not by RHC in the majority of hypoxic animals. Systolic RV pressure, PAP_m_, and vascular resistance increased, whereas PA compliance and distensibility decreased, in hypoxic animals. Thoracotomy caused cardiovascular depression, as evidenced by the reduced heart rate and flow velocity (Fig. 3).

**Table 3.** Hemodynamics

	Normoxia	Hypoxia	*P* Value
Heart rate, beats/min	331 ± 44	349 ± 24	0.26
Pulmonary trunk diastolic diameter, mm	2.7 ± 0.2	3.1 ± 0.3	<0.01*
Right ventricular systolic pressure, mmHg	16 ± 3	25 ± 2	<0.01*
Mean pulmonary arterial pressure, mmHg	11 ± 3	18 ± 3	<0.01*
Pulse pulmonary arterial pressure, mmHg	8 ± 1	13 ± 3	<0.01*
Right ventricular stroke volume, mm^3^	372 ± 61	312 ± 70	0.07
Cardiac output, ml/min	123 ± 25	109 ± 24	0.24
Right ventricular stroke work, ml⋅mmHg	4.2 ± 1.8	5.7 ± 2.1	0.15
Pulmonary acceleration time, ms	34 ± 7	26 ± 5	0.01*
Total pulmonary resistance, 10^3^ dyn⋅s⋅cm^−5^	7.5 ± 2.1	13.9 ± 4.6	0.01*
Pulmonary arterial compliance, mm^3^/mmHg	46.1 ± 8.7	23.1 ± 7.5	<0.01*
Pulmonary arterial distensibility, %/mmHg	5.7 ± 1.7	1.5 ± 0.7	<0.01*

Results are presented as means ± SD. Transthoracic echocardiography was performed on 8 normoxic animals and 10 hypoxic animals; right heart catheterization was performed on 10 normoxic animals and 11 hypoxic animals. Heart rate was obtained during echocardiography.

* *P* < 0.05.

**Fig. 4.**
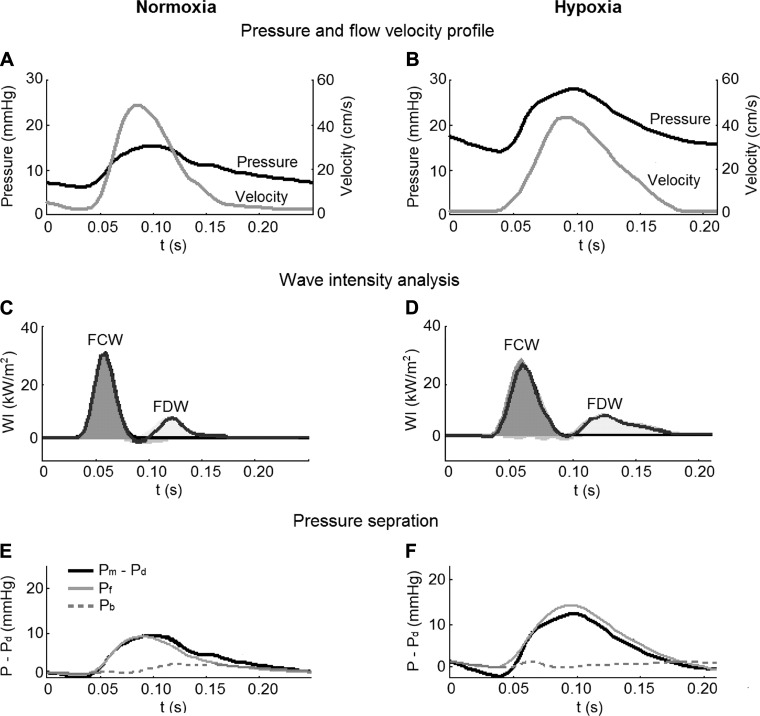
Pulmonary arterial wave propagation. Good quality velocity signals were obtained from 7 normoxic and hypoxic animals during right heart catheterization, and arterial wave analyses were performed. *A* and *B*: representative ensemble-averaged pressure (P) and flow velocity profiles from a normoxic and hypoxic rat (same animals as shown in Fig. 3). *C* and *D*: wave intensity analysis revealed a forward compression wave (FCW) and forward decompression wave (FDW) related to right ventricular ejection and relaxation, respectively. There were negligible backward waves present. The net wave intensity profile is highlighted in black. *E* and *F*: measured pressure [P_m_; minus diastolic pressure (P_d_)] was separated into forward (P_f_) and backward (P_b_) pressures. *t*, time.

**Fig. 5.**
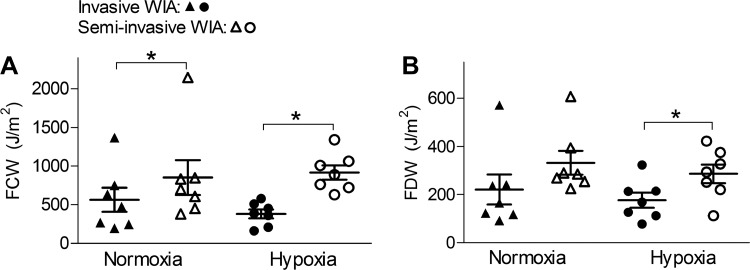
Wave intensities. Invasive wave intensity analysis (WIA) was performed using pressure and velocity data obtained by right heart catheterization, and semiinvasive WIA was performed using invasive pressure data combined with velocity data obtained by transthoracic echocardiography. In 5 normoxic animals and 6 hypoxic animals, both WIA methods were applied and pairwise comparison was performed. The energy densities of the forward compression wave (FCW; *A*) and forward decompression wave (FDW; *B*) derived from semiinvasive WIA were higher than their invasive counterparts (data presented as means ± SE). However, there were no significant differences in wave intensities between the two groups of animals, be it invasive or semiinvasive WIA. The same was observed for the peak intensities (not shown). *P < 0.05.

Invasive arterial wave analysis using RHC-derived pressure and velocity (Fig. 4) showed similar WIA and pressure separation patterns. The energy of the forward traveling waves related to ventricular contraction and relaxation was not detectably affected by hypoxia (Fig. 5). There was negligible backward WI (WRI < 5% in both groups). The P_b_-to-P_f_ ratio was 29.8 ± 7.2% in the normoxic group and similar in the hypoxic group (26.5 ± 6.0%). Semi-invasive WIA using RHC-derived pressure and TTE-derived velocity revealed larger wave energies compared with invasive WIA (Fig. 5) and a more prominent backward wave with a WRI of 8.3 ± 6.3% in the normoxic group and 9.5 ± 6.6% in the hypoxic group. The differences in wave energies and WRI between the two animal groups remained statistically insignificant, however.

#### Histological examination.

**Fig. 6.**
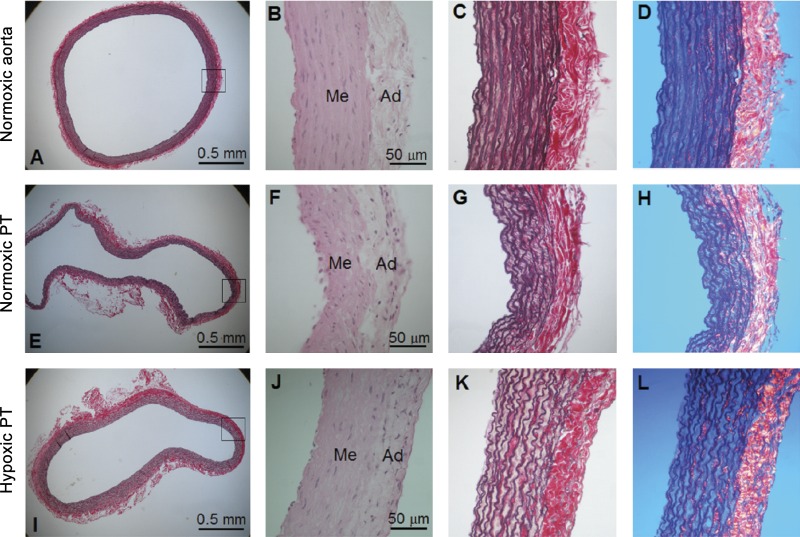
Representative histological sections were obtained from two normoxic and hypoxic animals. *A−L*: transverse histological sections of the aorta (*A−D*) and pulmonary trunk from a normoxic rat (*E–H*) and pulmonary trunk from a hypoxic rat (*I–L*). The appearance of the aorta from hypoxic animals (not shown here) was similar to normoxic animals. Images in *B*, *F*, and *J* were stained with hematoxylin and eosin; the rest were stained with resorcin, sirius red, and Weigert’s hematoxylin. Images in *C*, *G*, and *K* were photographed in bright field, wherein collagen fibers appear red and elastin fibers appear dark purple. Images in *D*, *H*, and *L* were photographed with a circular polarization filter, wherein collagen fibers appear yellow/orange/red and elastin fibers appear dark blue. Ad, adventitia; Me, media.

The PT was thinner than the aorta (Fig. 6, *A−H*). Elastin was mainly present as circumferentially arranged lamellae in the medial layer in both vessel types. In the aorta, elastin alternated between thick but nonwavy and thin wavy lamellae. In contrast, there were fewer elastic lamellae in the PT, and all lamellae were thin and wavy. Collagen fibers were mainly confined to the adventitia. After exposure to hypoxia, the histological appearance of the aorta was unchanged (not shown), whereas the medial and adventitial layers of the PT thickened (Fig. 6, *I−L*).

#### Mechanical properties.

**Fig. 7.**
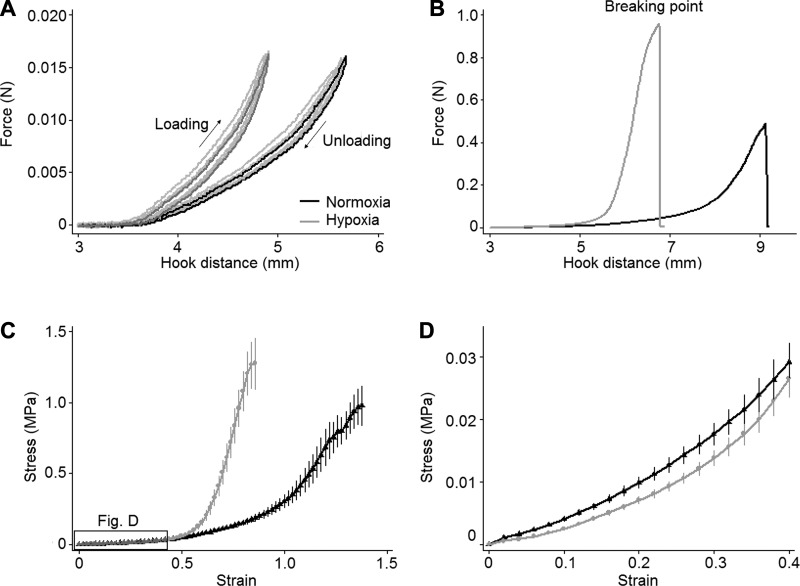
Force-deformation and stress-strain curves of the pulmonary trunk. *A*: loading-unloading cycles were performed five times for each artery ring (shown here for a representative ring from each animal group). The hysteresis loop from the last cycle is highlighted. *B*: a stretch test to rupture was performed on the same artery rings. *C* and *D*: the average stress-strain curve for the pulmonary trunk up to the breaking point (data presented as means ± SE; *C*) and at low-strain regions (with expanded scales; *D*) differed between the two animal groups.

**Fig. 8.**
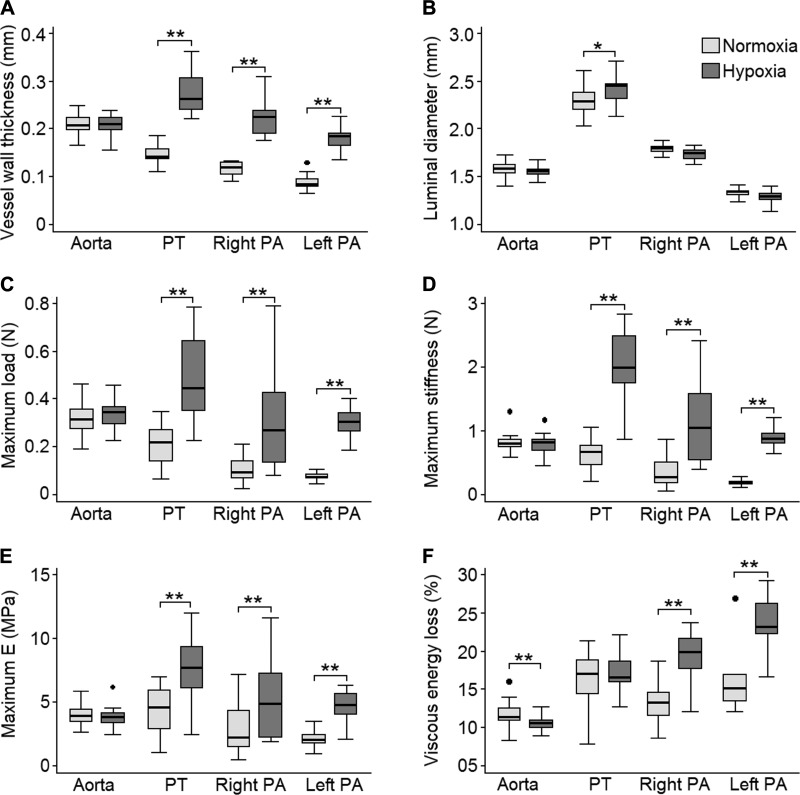
Significant changes were observed in the vessel dimensions and mechanical properties in hypoxic animals. *A−F*: vessel wall thickness (*A*), unstrained vessel luminal diameter (*B*), maximum load (*C*), maximum stiffness (*D*), maximum elastic modulus (*E*_max_; *E*), and viscous energy loss (*F*) of the aorta, pulmonary trunk (PT), and right and left pulmonary arteries (PAs). *P < 0.05; **P ≤ 0.001.

Stretch curves and dimensions and mechanical properties of the artery rings are shown in Figs. 7 and 8. The load-deformation curve of proximal PAs was shifted to the left in hypoxic animals (Fig. 7, *A* and *B*). The stress-strain curve was shifted to the left at high-strain regions, i.e., they had a smaller range of displacement, whereas they were mildly shifted downward in low-strain regions (Fig. 7, *C* and *D*).

Vessel wall thickness and the maximal tensile strength of proximal PAs, defined as the maximum load that artery rings could bear before rupturing, was increased in hypoxic animals (Fig. 8), whereas maximal stress was only significantly higher for the left PA (not shown). The maximal incremental arterial stiffness and elastic modulus also increased significantly (Fig. 8, *D* and *E*), whereas maximum strain significantly decreased by 1/3 (not shown). The energy loss due to wall viscosity in proximal PAs was significantly higher than in the aorta (Fig. 8*F*). In hypoxic animals, arterial wall viscosity significantly decreased in the aorta and increased in right and left PAs. No other mechanical changes were observed in the aorta.

**Fig. 9.**
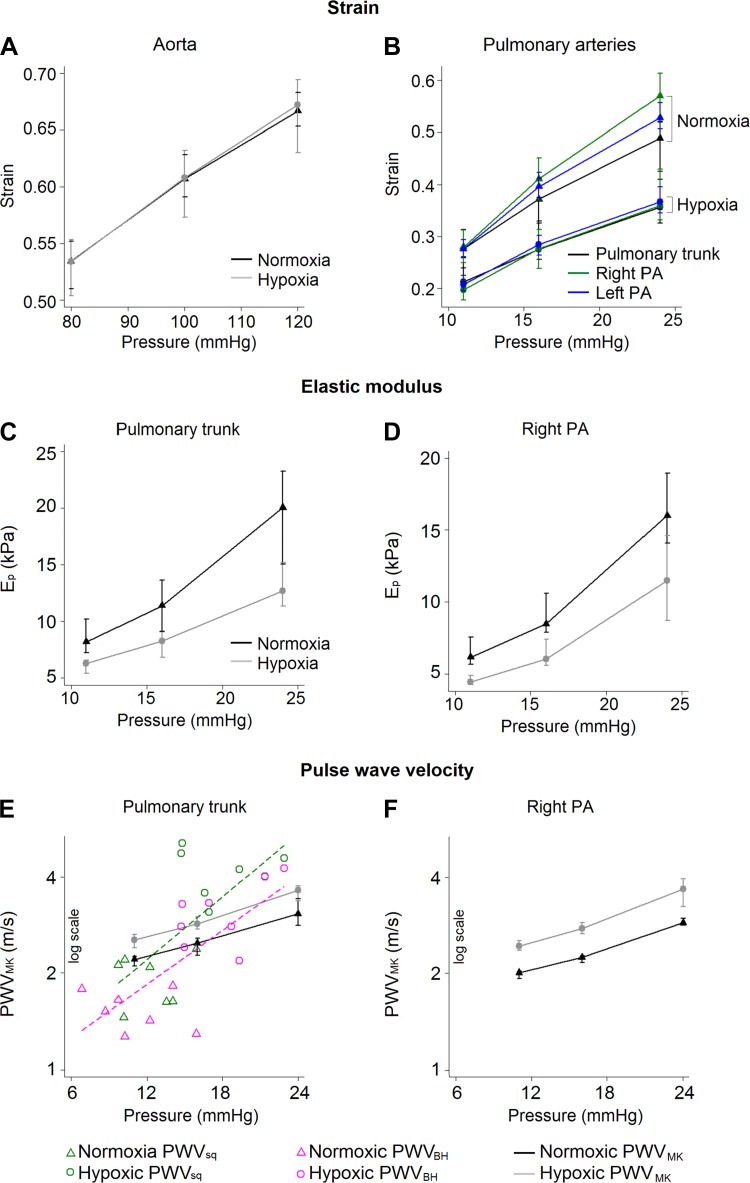
Mechanical properties at physiological distending pressures. *A* and *B*: strain-pressure curves for the aorta and pulmonary arteries (PAs). *C* and *D*: elastic modulus (E_p_) of the main and right PAs. *E* and *F*: empirical pulse wave velocity (PWV_MK_) was calculated using the Moens-Korteveg equation. Pulse wave velocity derived from the sum of squares (PWV_sq_) method and Bramwell and Hill equation (PWV_BH_) are plotted in *E*. Data are presented as medians (25–75% quartiles). The strain and *E*_p_ in PAs at each of the distending pressures (11, 16, and 24 mmHg) were significantly lower, wherease pulse wave velocity was significantly higher (*P* ≤ 0.01), in hypoxic rats.

At physiological distending pressures, vessel wall stress (not shown), strain, and *E*_p_ of proximal PAs were lower in hypoxic animals (Fig. 9). However, vessel wall stress and *E*_p_ at 24 mmHg in hypoxic animals were significantly higher than their counterparts at 11 mmHg in normoxic animals.

#### Pulse wave velocity.

Both PWV_sq_ and PWV_BH_ showed evidence of pressure dependence (higher with higher pressure; Fig. 9*E*). PWV_sq_ was 1.93 ± 0.35 m/s in normoxic animals and was increased 2.2-fold in hypoxic animals (4.18 ± 0.69 m/s). PWV_BH_ was significantly lower than PWV_sq_. PWV_BH_ increased 2.0-fold in the hypoxic group (1.54 ± 0.22 vs. 3.12 ± 0.72 m/s). PWV_MK_ showed clear evidence of pressure dependence in all three proximal PAs (not shown for left PA) and was significantly higher in the hypoxic group at each of the three distending pressures (Fig. 9, *E* and *F*). PWV_MK_ in the PT of hypoxic animals at 24 mmHg was 1.7-fold higher than its counterpart at 11 mmHg in normoxic animals. Furthermore, PWV_MK_ was significantly lower in right and left PAs compared with the PT within each animal group. A graphical comparison revealed a slight discrepancy between the in vivo- and ex vivo-derived PWV values (Fig. 9*E*), and the pressure dependence of in vivo values appeared steeper than for PWV_MK_. PWV_MK_ in the aorta also showed significant pressure dependence (∼5.0 m/s at 80 mmHg, ∼5.5 m/s at 100 mmHg, and ∼5.9 m/s at 120 mmHg for normoxic animals), and there were no significant differences between the two groups of animals.

#### ECM proteins.

**Fig. 10.**
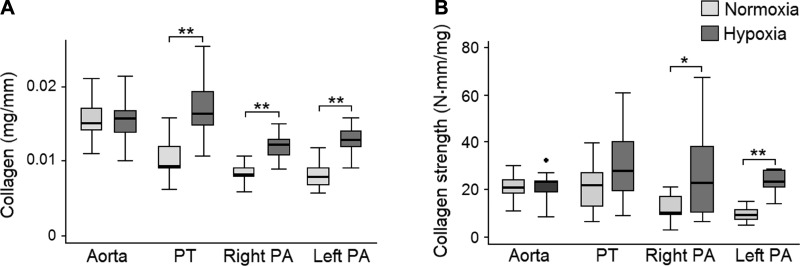
*A* and *B*: collagen content normalized to vessel circumference (*A*) and collagen strength defined as the maximum tensile strength per unit collagen (*B*) are shown for the aorta, pulmonary trunk (PT), and right and left pulmonary arteries (PAs). *P < 0.05; **P ≤ 0.001.

In the aorta, the absolute collagen content was similar in the two groups of animals (Fig. 10). However, the fractional collagen content was mildly reduced, whereas the fractional elastin content was significantly increased, in hypoxic animals, resulting in an increased elastin-to-collagen ratio (Table 4). The absolute and fractional collagen content as well as maximal tensile strength of collagen fibers in proximal PAs significantly increased in response to hypoxia, whereas the fractional elastin content and elastin-to-collagen ratio significantly decreased (Fig. 10 and Table 4). Compared the aorta, the elastin-to-collagen ratio was significantly lower in proximal PAs in both groups of animals. The fractional content of GAGs in proximal PAs increased significantly in response to hypoxia, and the fraction was significantly higher compared the aorta in both animal groups (Table 4). The fractional content of sulfated GAG was <1%.

**Table 4.** Extracellular matrix proteins

	Normoxia	Hypoxia	*P* Value
Aorta			
Elastin fraction, %	42.9 (42.1–43.1)	45.4 (45.3–45.7)	0.01*
Collagen fraction, %	27.7 (26.3–28.1)	26.2 (23.3–27.3)	0.08
Elastin-to-collagen ratio	1.52 (1.48–1.63)	1.73 (1.68–1.91)	0.03*
GAG fraction, %	3.61 (3.26–3.88)	4.04 (3.75–4.32)	0.12
GAG-to-collagen ratio	0.14 (0.11–0.14)	0.15 (0.14–0.19)	0.12
sGAG fraction, %	0.20 (0.14–0.24)	0.20 (0.18–0.24)	0.75
Pulmonary arteries			
Elastin fraction, %	23.0 (22.1–31.0)	17.3 (16.6–17.7)	0.03*
Collagen fraction, %	36.5 (35.7–36.7)	39.6 (38.5–40.6)	0.03*
Elastin-to-collagen ratio	0.63 (0.60–0.87)	0.43 (0.42–0.45)	0.03*
GAG fraction, %	5.81 (5.20–6.15)	6.36 (6.24–6.42)	0.03*
GAG-to-collagen ratio	0.16 (0.15–0.17)	0.16 (0.15–0.17)	0.72
sGAG fraction, %	0.46 (0.31–0.66)	0.44 (0.37–0.51)	1.0

Data are presented as medians (25–75% quartiles). GAG, glycosaminoglycan; sGAG, sulfated GAG. Excess artery tissues from 10 normoxic and hypoxic rats were gathered into three to five sample pools, and the protein content are expressed as the percentage of defatted dry weight.

* *P* < 0.05.

## DISCUSSION

We investigated the effect of chronic hypoxia on the mechanical properties and arterial wave propagation in proximal PAs. Collagen content, tensile strength per unit collagen, arterial stiffness, and viscous energy loss increased, whereas the elastin-to-collagen ratio and distensibility decreased. Pulmonary PWV was increased both in vivo and ex vivo, but hypoxia had little effect on arterial wave energies. In contrast to proximal PAs, aortic stiffness remained unchanged, whereas wall viscosity decreased.

### 

#### PA stiffness.

Hypoxia causes pulmonary vasoconstriction and increased hematocrit, leading to increased vascular resistance, elevated pressures, and arterial remodeling. In the early stages of PAH, a small increase in PVR can be associated with a considerable increase in arterial stiffness (49). The increase in PA stiffness will impair the Windkessel function, which could lead to RV dysfunction and promote damage in the distal vasculature. Aortic stiffness was unaffected by hypoxia, suggesting that the changes in proximal PAs may be chiefly related to the elevation of pressure secondary to hypoxic vasoconstriction in pulmonary arterioles rather than the effect of hypoxia per se or circulating factors released by the hypoxic lung.

The increase in the maximal elastic modulus observed in proximal PAs suggests that hypoxia not only induced hypertrophic remodeling but also a compositional change. Collagen content, the major determinant of the elastic behavior during supraphysiological loading, increases in proximal PAs in response to hypoxia (25, 43, 68). Therefore, the larger collagen fraction and increased tensile strength per unit collagen in hypoxic animals are likely to explain the higher elastic modulus. The mechanical quality of collagen is determined by the isoforms and organization the fibers, i.e., fiber thickness, distribution, cross-linking, and orientation, and further investigations are necessary to establish the mechanisms leading to the altered collagen tensile strength.

At low distending pressures, ∼50% of the load is carried by elastin fibers (27, 66). Interestingly, while the maximal elastic modulus was higher in hypoxic animals, the elastic modulus during physiological loading (*E*_p_) was decreased. Based on the elastin-to-collagen ratio, the absolute elastin content in proximal PAs is predicted to have increased by ∼20%. Indeed, several studies have reported elastin accumulation in PAs of hypoxic animals (25, 27, 45), which is consistent with the observed decrease in *E*_p_. However, since hypoxic animals experience a higher distending pressure, they do not operate on the same portion of the nonlinear pressure-strain curve and the tension is transferred to less-extensible collagen fibers. Therefore, in vivo, *E*_p_ of PAs in hypoxic animals is higher than normoxic animals.

Whether the increased GAG content contributes to vessel stiffening is unclear. The main classes of GAGs found in the vessel wall include chondroitin sulfate, dermatan sulfate, heparan sulfate, and hyaluronan (31), all of which contain uronic acid. As evidenced by the low fractional content of sulfated GAG, the main constituent of GAG in proximal PAs must be hyaluronan, a nonsulfated GAG. Overexpression of hyaluronan has been shown to increase the mechanical strength and stiffness of the aorta (7), whereas hyaluronan digestion had no effect on the mechanical properties (33, 44).

#### Arterial viscoelasticity.

PAs demonstrate viscoelastic behavior. In hypoxic animals, energy dissipation due to viscous behavior was increased in right and left PAs. This may be due to one or more compositional changes in the artery, including increased collagen content (66, 67), increased GAG content, and/or altered smooth muscle cell tone (11, 47, 67), although the contribution from smooth muscle cells was likely to have been underestimated due to the experimental setup in the present study.

Altered arterial viscosity may alter the dynamic interaction between the RV and pulmonary circulation. The increased loss in arterial pulse wave energy augments the dynamic RV afterload (65). However, the viscous losses will also reduce the energy of any reflected compression waves and thus reduce the dynamic RV afterload (18). Thus, the exact implication of the altered viscosity on the net ventricular load is unclear.

Proximal PAs displayed a higher viscosity compared with the aorta, consistent with the higher collagen and GAG fractions. This may contribute to the lower oscillatory to total ventricular power fraction in the left-sided system: ∼10% (38, 42) versus 25–30% in the right-sided system (29, 35, 48). Interestingly, the viscous energy loss decreased in the aorta of hypoxic animals, which may be attributed to the mildly increased elastin content. Thus, altered elastin metabolism in response to hypoxia may be a more widespread feature. The increased elastin content did not affect aortic *E*_p_, however. In the aorta, the elastic fiber engagement plateaus around a strain of 0.2 (9), lower than that of PAs (27, 66), perhaps due to the less “wavy” nature of elastin lamellae in the aorta. At physiological pressures, the aorta operates at a higher strain (> 0.5), which leads to a substantial recruitment of collagen fibers, the content of which was unaffected by hypoxia.

#### Pulse wave velocity.

PWV, in vivo and ex vivo, increased in hypoxic animals. PWV_sq_, calculated from the pressure and velocity measurements, was higher than PWV_BH_, which was determined by the pulsatile changes in pressure and luminal vessel area. This is consistent with a previous study (53), where PWV derived from the PU-loop method, which is related to PWV_sq_, was consistently higher than PWV_BH_. By calculating PWV_MK_ at three different distending pressures ex vivo, we have demonstrated that the increased PWV in hypoxic animals was not only due to increased pressure but also related to structural or constitutive changes in the vessel wall. PWV_sq_, PWV_BH_, and PWV_MK_ all displayed pressure dependence, which appeared more marked in vivo than ex vivo.

While the tensometer setup in the present study allows fine control of the circumferential stretch and is unaffected by the anesthesia and volume status of the animal, it does not take into account the influence of sympathetic activation and smooth muscle cell tone nor the effect of longitudinal vessel wall tension. Sympathetic activation increases in pulmonary hypertension (63) and in response to hypoxia (19). After direct sympathetic stimulation in intact dogs, PA compliance decreases (24, 59), and this may explain the larger increase in arterial stiffness in vivo. Vasoconstriction reduces vessel diameter and thus reduces collagen fiber engagement. However, increased vascular tone may also lead to an elevated elastic modulus in both the elastin and collagen-dominant strain ranges (26). Thus, the contribution of smooth muscle cell tone to proximal arterial stiffness is not easily predictable (43, 67).

#### Arterial wave propagation.

To the best of our knowledge, this is the first study to perform invasive pulmonary WIA in a rodent model. We observed similar wave intensities in the two groups of animals; notably, there was no backward wave of large intensity. Previous studies in healthy humans have shown little wave reflection in the PA, whereas in patients with PAH (30, 57) and dogs exposed to acute hypoxia (21, 40), a large midsystolic backward compression wave has been observed. By performing pressure separation based on the characteristic impedance, the P_b_-to-P_f_ ratio was estimated to 20–30% in normoxic and hypoxic mice (51, 60), comparable to the present study. The minimal wave reflection in both groups of animals may be attributable to a matched energy transmission between the proximal and distal vasculature and/or the attenuation of discrete backward wave transmission due to the extensive branching system of the pulmonary vasculature (2). The more viscous proximal PAs and increased blood viscosity due to the increased hematocrit in hypoxic animals may also increase wave energy dissipation.

It is also possible that detection of wave reflection was influenced by methodological limitations. Flow velocity acquired by RHC was significantly lower than its noninvasive TTE counterpart and the midsystolic notching was lost. Differences between the invasive and noninvasive measurements can partially be explained by thoracotomy (17, 32) and perhaps by the use of sevoflurane plus nitrous oxide anesthesia. However, inhaled nitrous oxide does not have the same pulmonary vasodilatory effect as inhaled nitric oxide. While inhaled nitric oxide is a potent and selective pulmonary vasodilator that can reverse hypoxic pulmonary vasoconstriction (23), there is no evidence that inhaled nitrous oxide acts in the same way (20). The intravascular catheter used in this study must also be considered a limitation: the diameter of the catheter (0.36 mm) was relatively large (10–15% of the PT diameter) and could lead to disturbed flow adversely affecting WI estimates. WIA using RHC-derived pressure combined with TTE-derived velocity resulted in WIs that were larger with discernable, albeit small, reflections that did not differ between normoxic and hypoxic animals. However, importantly, RHC and TTE were not performed simultaneously and were measured under open- and close-chested conditions, respectively; hence, changes in TTE-derived velocity may not correspond to the changes in RHC-derived pressure and combining them for WIA may result in altered or artifactual waves.

Multisensor catheters minimize the uncertainties related to spatial and temporal alignment of the pressure and velocity signals (56), and we have demonstrated that invasive pulmonary WIA in the rat is feasible. However, given the necessity of thoracotomy and the potential influence of the relatively large caliber catheter, and given that good quality velocity data could not be obtained in 33% of the animals, this is a technically challenging technique with significant limitations. In recent years, specially designed pressure catheters have made it possible to perform PA catheterization in close-chested rats (14, 61), which, together with simultaneous TTE, may enable less invasive application of pulmonary WIA. In addition, completely noninvasive ultrasound application of WIA by tracking the change in vessel wall diameter as a surrogate marker for pressure changes is feasible in the murine aorta and carotid artery (15, 16). Whether this method can be applied in the PA requires further investigation.

#### Limitations.

We used only male animals as the effect of chronic hypoxia on female rats are not well established. In contrast to the newly developed SuHx rodent model based on chronic hypoxia in combination with VEGF inhibition, exposure to chronic hypoxia alone only causes a mild to moderate pressure elevation in rodents (54). The comparatively low pressures recorded in the present study may also be related to cardiovascular depression caused by thoracotomy and anesthesia and oxygen inhalation. In addition, inaccurate zero calibration may underestimate the pressure estimates (described in detail in the appendix).

The shortcomings of open-chested RHC have already been discussed. There are also several limitations associated with TTE. B-mode measurement of vessel dimensions in small animals can yield substantial spatial errors, leading to errors in the cardiac output and arterial distensibility estimates.

As discussed above, in vivo and ex vivo experiments were performed on different subsets of animals. This precludes us from performing pairwise comparison and correlation analyses. Mechanical testing was performed on thawed frozen artery rings in calcium- and magnesium-free buffer, and, thus, the influence of sympathetic activation and smooth muscle cell tone was neglected. Studies on the impact of the freeze/thaw cycles on the passive mechanical properties of porcine arteries have shown conflicting results (41, 64); however, freezing probably affects normoxic and hypoxic vessels to the same extent.

Due to their small size, it was not possible to determine the elastin content in the artery rings used for mechanical testing, and the elastin content was measured in surplus arteries. By measuring the absorbance of uronic acid, which is present in all GAGs in the vessel, the fractional contribution from the different classes of GAG cannot be estimated. Moreover, the absorbance property differs depending on the class of GAG (10).

#### Conclusions.

Proximal PAs of hypoxic rats have altered ECM protein composition, increased collagen tensile strength, and altered mechanical properties. Exposure to hypoxia is associated with higher stiffness of the PA in vivo and ex vivo. There was no convincing evidence of altered WI or increased wave reflection in hypoxic animals, although methodological issues may limit this conclusion. Understanding PA wave propagation and the structural and mechanical properties provides novel insights into the impact of altered dynamic afterload on the RV in PAH, which could ultimately uncover new treatment targets.

## GRANTS

J. Su received support from European Respiratory Society ERS PAH Long-Term Research Fellowship LTRF 2013–2183 and Aarhus University Graduate School. A. D. Hughes received support from a National Institute for Health Research Biomedical Research Centre Award to University College London Hospital and British Heart Foundation Grants PG/15/75/31748, CS/15/6/31468, and CS/13/1/30327. U. Simonsen received funding from the Novo Nordisk Foundation and Manufacturer Vilhelm Pedersen and Wife’s Scholarship. The funders played no role in the preparation of the manuscript or decision to publish.

## DISCLOSURES

No conflicts of interest, financial or otherwise, are declared by the author(s).

## AUTHOR CONTRIBUTIONS

J.S., A.D.H., and U.S. conceived and designed research; J.S., C.C.L., and C.C.D. performed experiments; J.S., K.H.P., N.M.D., and C.C.D. analyzed data; J.S., A.D.H., K.H.P., C.C.D., and U.S. interpreted results of experiments; J.S. prepared figures; J.S. drafted manuscript; J.S., C.C.L., A.D.H., K.H.P., N.M.D., C.C.D., and U.S. edited and revised manuscript; J.S., C.C.L., A.D.H., K.H.P., N.M.D., C.C.D., and U.S. approved final version of manuscript.

## APPENDIX

Pressure and velocity data were processed using custom-written Matlab software (v2015a, MathWorks). Pressure and velocity signals obtained by RHC were ensemble averaged using maximum dP/d*t* as a fiducial marker for each cardiac beat. Signals were smoothed, and the first derivative of the data was calculated using a Savitzky-Golay differentiating filter (second order polynomial fit, window size 5). Due to the occurrence of pressure drift, ensemble-averaged RV pressure was calibrated by setting RV diastolic pressure to 0 mmHg and PA pressure was calibrated by setting PA systolic pressure to RV systolic pressure. PAP_m_ was taken as the arithmetic mean over the cardiac cycle. The zero calibration may lead to an underestimation of RV systolic pressure and PAP_m_. This does not affect pulse pressure or dP, however. To apply WIA, hardware-related delay between the signals was corrected by shifting the ensemble-averaged velocity data until the start of the upslope of the velocity and pressure waveforms were aligned.

Velocity data acquired by TTE were extracted at a sampling rate of 200 Hz (to match RHC-derived data) by tracking the Doppler flow envelope using a screenshot of the pulsed-wave Doppler image. Signals were subsequently ensemble averaged using maximum d*U*/d*t* as a fiducial marker. To apply WIA using RHC-derived pressure combined with TTE-derived velocity, the ensemble-averaged velocity and pressure waveforms were synchronized by aligning the upslope (early systole) of the ensemble-averaged velocity and pressure waveforms. This assumes that wave reflection is negligible in this period and is a key assumption of separated WI (and impedance analysis). The measurements were not obtained simultaneously, and therefore the heart rate varied. To overcome this problem, diastole was truncated to achieve a consistent cycle durations for both waveforms.

Separated (forward and backward) pressure waveforms (P_f_ and P_b_) were obtained by integrating their derivatives: dP_f_/d*t* and dP_b_/d*t* (calculated using *Eq. 7*), where the integration constant was taken to be zero. Therefore, the sum of P_b_ and P_f_ is the measured pressure minus minimum measured pressure (i.e., diastolic pressure).
